# The relationship between alexithymia, emotion regulation, psychological resilience, and self-management behaviors in kidney transplant recipients: a multicenter structural equation modeling study

**DOI:** 10.3389/fpsyt.2026.1909478

**Published:** 2026-08-17

**Authors:** Meiqian Chen, Lili Zou, Lianyu Lou, Zhiying Lei, Junyi Huang, Xiang Fu, Yanjuan Lin

**Affiliations:** 1 Organ Transplantation Center, Fujian Medical University Union Hospital, Fuzhou, China; 2 Department of Laoshan Nursing Unit of Kidney and Lung Transplantation, Affiliated Hospital of Qingdao University, Qingdao, China; 3 Institute of Transplantation Medicine, The Second Affiliated Hospital of Guangxi Medical University, Nanning, China; 4 Heart Medical Center, Fujian Medical University Union Hospital, Fuzhou, China; 5 Department of Nursing, Fujian Medical University Union Hospital, Fuzhou, China

**Keywords:** alexithymia, emotion regulation, psychological resilience, self-management, kidney transplantation, structural equation modeling

## Abstract

**Background:**

Effective self-management behaviors can improve outcomes in kidney transplant recipients. Although existing evidence has shown that alexithymia is negatively associated with self-management behaviors, the underlying mechanisms remain unclear. This study aimed to explore the relationships among the alexithymia, emotion regulation, psychological resilience and self-management behaviors in kidney transplant recipients.

**Methods:**

This multicenter cross-sectional study recruited kidney transplant recipients who attended follow-up visits at three major tertiary hospitals in northern and southern China between January and March 2026. A general demographic questionnaire, the Perth Alexithymia Questionnaire, the Emotion Regulation Questionnaire, the Kidney Transplant Psychological Resilience Scale, and the Kidney Transplant Self-Management Scale were used for data collection. The relationships among alexithymia, emotion regulation, psychological resilience, and self-management were examined using correlation analysis, linear regression, and structural equation modeling.

**Results:**

A total of 299 kidney transplant recipients were included in the analysis. The structural equation model demonstrated a good fit to the data (CFI = 0.949 - 0.955; RMSEA = 0.077 - 0.085). Alexithymia was directly associated with self-management in two parallel structural equation models (direct effect = −0.129 in the cognitive reappraisal model; −0.119 in the expressive suppression model). Emotion regulation strategies, including cognitive reappraisal and expressive suppression, mediated the association between alexithymia and self-management (indirect effect = −0.030 and −0.041, respectively). Furthermore, a chain mediating pathway through emotion regulation strategies and psychological resilience was identified (indirect effect = −0.028 and −0.067, respectively).

**Conclusion:**

Alexithymia is both directly and indirectly associated with poorer self-management behaviors in kidney transplant recipients, with emotion regulation strategies and psychological resilience serving as significant mediators. These findings provide preliminary directions for optimizing personalized self-management support following kidney transplantation.

## 1 Introduction

Kidney transplantation is the most effective treatment for end-stage renal disease, with post-transplant survival rates of 91.27%, 86.46%, 81.17%, and 78.15% at 1, 3, 5, and 10 years, respectively (1). After transplantation, recipients are required to take immunosuppressive agents regularly for life and remain under the long-term dual risk of rejection and infection, which can easily lead to psychological stress. Persistent psychological stress may disrupt sleep rhythms, induce endocrine disturbances, and increase pro-inflammatory cytokine levels, thereby elevating the risks of rejection and infection in kidney transplant recipients (2). The complexity of post-transplant treatment regimens, difficulties in adapting to a new lifestyle, and concerns and fear regarding the transplanted organ together underscore the need for kidney transplant recipients to maintain good self-management behaviors. A study has shown that 28.7% of kidney transplant recipients exhibit poor self-management, which is associated with a 2.66-fold higher risk of death, a 6.44-fold higher risk of graft loss, and a 2.28-fold higher risk of rejection (3). Poor adherence to oral medication has been associated increase variability in tacrolimus blood concentrations, which may be associated with the coexistence of polyomavirus nephropathy and acute rejection (4). To prevent rejection, reduce infection and prolong graft survival, kidney transplant recipients must improve their self-management.

Self-management in kidney transplant recipients is a multidimensional concept encompassing treatment regimen management, emotional processing, and adaptation to new roles. However, current research has predominantly focused on treatment regimen management, with limited attention to emotional adaptation and role adjustment (5). A systematic review of 27 intervention studies found inconsistent benefits for emotional outcomes such as anxiety and depression, while studies on role adaptation—including social support, interpersonal relationships, and comfort—were exceedingly rare (6). This imbalance hinders a comprehensive understanding of self-management and the development of tailored interventions.

The potential for post-transplant complications, such as rejection and infection, can impose substantial psychological stress on recipients. If suppressed emotions in kidney transplant recipients are not effectively relieved, they may manifest through somatization (7). This process may be significantly influenced by alexithymia, which is characterized by an externally oriented thinking style (expressed through somatization symptoms), difficulty identifying feelings, and difficulty describing feelings (8). Alexithymia is not uncommon among kidney transplant recipients, with a prevalence of approximately 23% (7, 9). A study has shown that higher levels of alexithymia are associated with poorer treatment adherence, with affected patients being more likely to exhibit non-adherent behaviors such as missed or forgotten doses (9). Patients with alexithymia tend to perceive their quality of life more negatively, particularly in the domain of mental health. Such negative perceptions may weaken patients’ motivation and initiative to engage in self-management. According to stress and coping theory, individuals’ responses to stressors are shaped by their cognitive appraisal and coping resources (10). Alexithymia, as a deficit in emotional processing, may act as a stressor that triggers maladaptive cognitive appraisal and weakens coping capacity, thereby negatively influencing health behaviors.

The process by which individuals activate goals to influence the generation, experience and expression of their emotions is known as emotion regulation (11). This process can be achieved through a variety of strategies, among which expressive suppression and cognitive reappraisal are the two most common and extensively studied approaches. Study has shown that kidney transplant recipients who lack effective emotion regulation abilities and have difficulty regulating negative emotions may experience impaired renal function, as reflected by elevated creatinine levels (9). Patients with higher levels of alexithymia often have difficulty identifying and describing their emotions. Consequently, they may find it difficult to activate a clear goal aimed at changing their emotional state. Even when emotions have already caused considerable distress, the lack of a clear regulatory goal may leave them unsure of how to respond, ultimately leading to failure in self-management behaviors.

After transplantation, patients must also cope with complications, disruption of social roles and financial burden (12). Coping with these chronic stressors requires kidney transplant recipients to possess psychological resilience, which is the capacity to respond effectively to stress, positively transform negative emotions and overcome crises (13). Enhanced psychological resilience can promote both physical and mental health in kidney transplant recipients (14). As a component of self-management, medication adherence has also been linked to higher resilience (13). When psychological resilience is depleted and cannot be effectively replenished, the resources required to maintain effective self-management may become insufficient, potentially resulting in poorer self-management.

**Figure 1:**
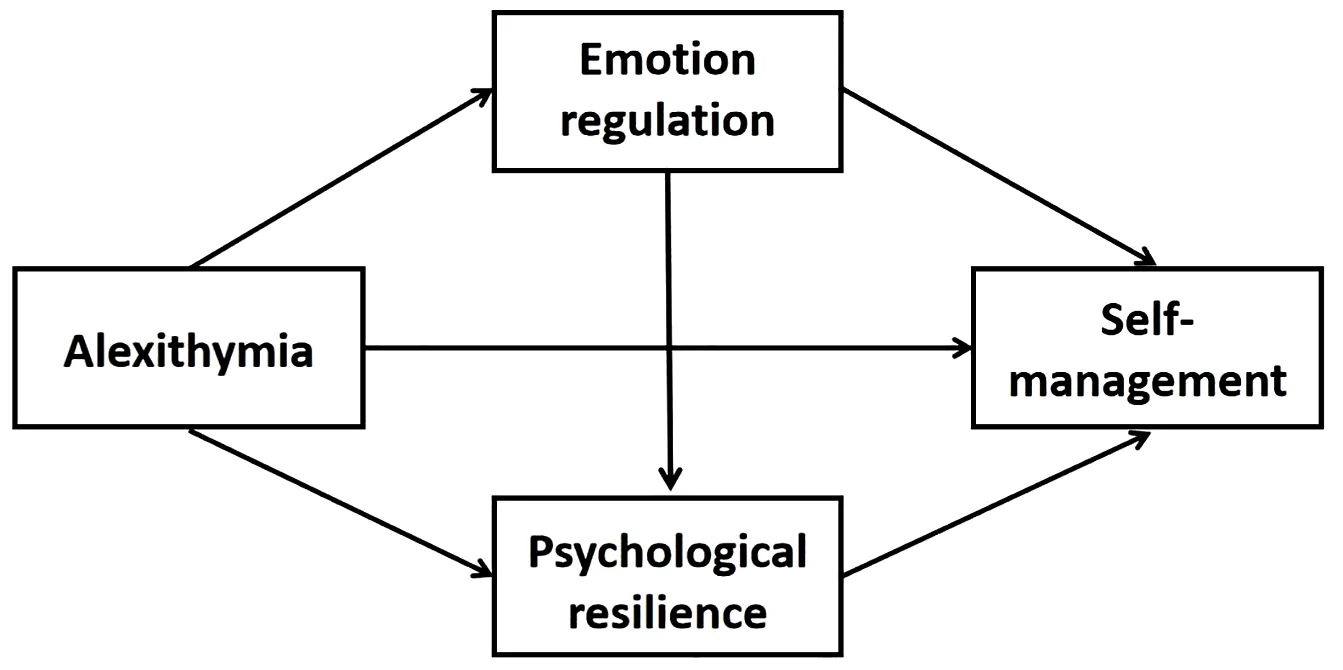
Hypothesized model of the study based on stress and coping theory.

According to stress and coping theory, alexithymia may act as a stressor that triggers biased cognitive appraisal, weakens emotion regulation capacity and resilience-related coping resources, and thereby negatively affects self-management behaviors in kidney transplant recipients (10). On this basis, we hypothesized that the relationship between alexithymia and self-management in kidney transplant recipients is mediated by emotion regulation and psychological resilience (**Figure 1**). To the best of our knowledge, no study has systematically examined these four variables within a single analytical framework. A recent study examined alexithymia and somatic symptoms in patients with chronic kidney disease (CKD) and highlighted the role of clinical and psychosocial factors (15). However, that study focused on the pre-transplant CKD population and did not examine the mediating mechanisms linking alexithymia to self-management behaviors. In contrast, the present study specifically targets kidney transplant recipients and investigates the mediating roles of emotion regulation strategies and psychological resilience, thereby extending the evidence base to the post-transplant context. This gap limits a deeper understanding of the roots of self-management difficulties in kidney transplant recipients and hinders the development of personalized psychological interventions. Therefore, the direct and indirect effects of alexithymia on self-management in kidney transplant recipients were examined in this study using structural equation modeling, with a focus on the mediating roles of psychological resilience and emotion regulation (cognitive reappraisal and expressive suppression). The findings may provide a solid theoretical basis for nurses to develop individualized psychological support and self-management interventions, and have significant theoretical and practical implications for enhancing kidney transplant recipients’ quality of life and long-term prognosis.

## 2 Materials and methods

### 2.1 Study design

The design of this study was cross-sectional.

### 2.2 Participants

This study used a convenience sampling method to recruit kidney transplant recipients who attended outpatient follow-up at three tertiary hospitals from January to March 2026. The inclusion criteria were as follows: (1) aged 18 years or older; (2) first kidney transplantation; and (3) ability to understand and communicate, and provision of informed consent. The exclusion criteria were: (1) severe postoperative complications; (2) cognitive impairment or failure of other vital organs; and (3) receipt of combined multi-organ transplantation.

The required sample size was determined using the N:q ratio, a commonly recommended SEM-specific criterion (16). Each structural equation model included approximately 35 free parameters. Based on the recommended N:q ratio of at least 5:1, the minimum required sample size was 175.

### 2.3 Measurement tools

The key constructs in this study were operationalized as follows. Alexithymia was defined as difficulty identifying and describing emotions, along with an externally oriented thinking style, in kidney transplant recipients. Consistent with the instrument used, the difficulty in identifying and describing feelings pertains to both positive and negative emotions. It was measured using the Perth Alexithymia Questionnaire. Emotion regulation was operationalized in terms of two specific strategies: cognitive reappraisal and expressive suppression. Cognitive reappraisal refers to reframing the meaning of an emotion-eliciting event. Expressive suppression refers to inhibiting outward emotional expression. Both strategies were assessed using the Emotion Regulation Questionnaire. Psychological resilience was conceptualized as the capacity of kidney transplant recipients to maintain positive adjustment. This capacity is reflected in a positive attitude, medical management, social support, and healthy habits. It was measured using the Kidney Transplant Psychological Resilience Scale. Self-management behavior was defined as daily practices encompassing dietary management, treatment adherence, physical activity, and psychosocial coping. It was assessed using the Kidney Transplant Self-Management Scale.

#### 2.3.1 General demographic questionnaire

This questionnaire comprised 12 items, including age, gender, educational level, place of residence, marital status, insurance status, financial burden, time since transplantation, occupation, diagnosis, hypertension and the age-adjusted Charlson Comorbidity Index (17).

#### 2.3.2 Perth alexithymia questionnaire

The Perth Alexithymia Questionnaire was developed by Preece et al. in 2018 and later translated into Chinese by Niu et al. (18). The scale consists of 24 self-report items across five dimensions: negative difficulty identifying feelings, positive difficulty identifying feelings, negative difficulty describing feelings, positive difficulty describing feelings, and externally oriented thinking. Each item is scored on a 7-point Likert scale ranging from 1 (“strongly disagree”) to 7 (“strongly agree”). Total score range from 24 to 168. Higher scores indicate higher levels of alexithymia. The Chinese version demonstrated good reliability, with a Cronbach’s α of 0.953. The Cronbach’s α for this research was 0.956.

#### 2.3.3 Emotion regulation questionnaire

The Emotion Regulation Questionnaire was developed by Gross and John in 2003 and later translated into Chinese by Chen et al. (19). The questionnaire comprises ten items across two dimensions: cognitive reappraisal and expressive suppression. Each item is scored on a 7-point Likert scale ranging from 1 (“strongly disagree”) to 7 (“strongly agree”). Higher scores indicate more frequent use of the corresponding strategy. The Cronbach’s α for expressive suppression and cognitive reappraisal in the Chinese translation were 0.76 and 0.87, respectively. The equivalent Cronbach’s α in this study was 0.790 and 0.858, respectively.

#### 2.3.4 Kidney transplant psychological resilience scale

This scale was developed by Chung et al., and the Chinese version was adapted by Tang et al. (20). It consists of four dimensions: positive attitude, medical management, social support, and healthy habits. All items are positively scored on a 4-point Likert scale ranging from 1 (“strongly disagree”) to 4 (“strongly agree”). Higher scores indicate greater psychological resilience, with total scores ranging from 22 to 88. The Cronbach’s α of the scale was 0.944, indicating good internal consistency. The Cronbach’s α in this study was 0.930.

#### 2.3.5 Kidney transplant self-management scale

The Kidney Transplant Self-Management Scale was developed by Zhuang et al. (21). This scale is used to evaluate kidney transplant recipients’ self-management practices and consists of 28 items. The scale comprises four dimensions: dietary management, treatment management, physical activity management, and psychosocial management. Each item is assessed on a 4-point Likert scale, with total scores ranging from 28 to 112. Higher scores indicate better self-management. Scores of > 90, 68 - 90, and < 68 represent good, moderate, and poor self-management, respectively. The scale has demonstrated strong validity, with a Cronbach’s α of 0.857. The Cronbach’s α for this study was 0.929.

### 2.4 Data collection

Questionnaires were administered to outpatients who had received a kidney transplant more than three months prior. The purpose, content, procedures, and significance of the study were explained to the patients. After providing informed consent by signing the consent form, they completed the questionnaire in a face-to-face setting. Before data collection, all researchers received standardized training to ensure consistency in the study procedures, instructions, questionnaire completion guidelines, and quality control requirements. During the survey, researchers used standardized instructions to explain the completion procedures and precautions, answered participants’ questions individually, and conducted one-on-one interviews with objective recording for those unable to complete the questionnaire independently. Upon completion, each questionnaire was immediately checked on-site for completeness and logical consistency. Any omissions or inconsistencies were promptly corrected. Strict quality control procedures were implemented throughout to ensure that the data were accurate, reliable, and valid.

### 2.5 Statistical analysis

Data were entered using Epidata, and statistical analyses was performed using R software (4.4.1) after data export. First, normality was assessed using skewness and kurtosis. Continuous data with a normal distribution are presented as mean ± standard deviation (x̄ ± s), whereas continuous variables with a non-normal distribution are presented as medians (interquartile range). Categorical variables are presented as frequencies and percentages (%). An independent-samples t-test was used to compare two groups with normally distributed data and homogeneous variances, and one-way analysis of variance was used to compare multiple groups. When the assumption of homogeneity of variance was not met, Welch’s test was employed. The Mann-Whitney U test was used to compare two groups with non-normally distributed data, whereas the Kruskal-Wallis test was used to compare multiple groups. Spearman’s correlation was used for non-normally distributed data, whereas Pearson’s correlation was used for normally distributed data. Second, the associations among alexithymia, emotion regulation, psychological resilience, and self-management were examined using linear regression analysis.

Finally, structural equation modeling was performed using the lavaan package in R. To construct the latent variable indicators for structural equation modeling, we adopted an item-parceling approach based on the dimensional structure of each scale. The dimension scores of each scale were used as observed indicators for their respective latent constructs. This approach was chosen because it reduces the number of estimated parameters, improves the sample size-to-parameter ratio, and enhances model stability and statistical power (22). Additionally, dimension-level parcels yield more reliable and normally distributed indicators than individual items. The dimensional structures of all scales have been well-established in prior validation studies, providing a strong theoretical basis for parceling at the dimension level.

Cognitive reappraisal and expressive suppression were examined in two separate structural equation models rather than a single integrated model. This decision was based on conceptual considerations. Conceptually, cognitive reappraisal and expressive suppression represent distinct emotion regulation strategies with different theoretical mechanisms. Cognitive reappraisal involves reframing the meaning of an emotion-eliciting event, whereas expressive suppression involves inhibiting outward emotional expression. Including both strategies as parallel mediators in a single model would have substantially increased model complexity. Moreover, the shared variance between them would be partitioned in a way that obscures each strategy’s unique contribution. Therefore, two parallel models were specified to examine the mediating roles of each emotion regulation strategy separately, consistent with previous studies that have adopted similar approaches when examining distinct mediating pathways (23). Model fit was assessed using the following indices: chi-square/degrees of freedom ratio (χ²/df ≤ 3), goodness-of-fit index (GFI ≥ 0.9), Tucker-Lewis index (TLI ≥ 0.9), normed fit index (NFI ≥ 0.9), comparative fit index (CFI ≥ 0.9), and root mean square error of approximation (RMSEA ≤ 0.1) (24). Model estimation was performed using the maximum likelihood (ML) estimator. The indirect effects were evaluated using bootstrap confidence intervals based on 1,000 resamples. Listwise deletion was applied for missing data.

## 3 Results

### 3.1 Common method bias

Common method bias was assessed using Harman’s single-factor test. The unrotated exploratory factor analysis yielded 16 factors with eigenvalues greater than 1, which together accounted for 68.13% of the total variance. The first factor explained 32.96% of the variance, which falls below the 40% threshold (25). This result suggests that common method bias did not substantially affect the study findings.

### 3.2 Participant characteristics

Of the 305 questionnaires distributed, 299 were returned as valid, yielding a valid response rate of 98.03%. The sample comprised 185 men (61.9%) and 114 women (38.1%). Among the participants, 168 (56.2%) were aged 18–44 years, 99 (33.1%) were aged 45–59 years, and 32 (10.7%) were aged 60 years or older. Fifty participants (16.7%) were 2–3 years post-transplantation, and 92 (30.8%) were more than 3 years post-transplantation. A total of 233 participants (77.9%) were married, 200 (66.9%) lived in urban areas, and 223 (74.6%) had employee medical insurance. The primary kidney disease was unspecified in 139 participants (46.5%). In total, 137 participants (45.8%) had a university education or above, 135 (45.2%) were unemployed, 224 (74.9%) had hypertension, and 112 (37.5%) perceived their financial burden as heavy. As shown in **Table 1**, the mean comorbidity score was 0.62 ± 0.90. Self-management scores differed significantly by gender and perceived financial burden (p < 0.05).

**Table 1:** General characteristics of kidney transplant recipients.

Variables	Sample size(%) Mean ± SD	Self-management behaviors score	Statistic	p value
Gender				
Male	185 (61.9%)	79.69 ± 12.64	-3.393^a^	0.001
Female	114 (38.1%)	84.64 ± 11.62		
Age group				
18 ≤ age < 45	168 (56.2%)	80.54 ± 12.42	2.580^b^	0.077
45 ≤ age < 60	99 (33.1%)	81.94 ± 12.63		
age ≥60	32 (10.7%)	85.91 ± 11.61		
Duration after operation (months)				
3 ≤ time ≤ 12	88 (29.4%)	83.47 ± 11.85	2.042^b^	0.108
12 < time ≤ 24	69 (23.1%)	82.91 ± 13.36		
24 < time ≤ 36	50 (16.7%)	80.42 ± 12.93		
> 36	92 (30.8%)	79.39 ± 11.91		
Marital status				
Single	60 (20.1%)	79.20 ± 11.69	2.305^b^	0.102
Married	233 (77.9%)	82.35 ± 12.65		
Divorce	6 (2.0%)	75.33 ± 10.05		
Residence				
Urban	200 (66.9%)	81.20 ± 12.16	-0.739^a^	0.461
Rural	99 (33.1%)	82.33 ± 13.12		
Insurance type				
Employee Medical Insurance	223 (74.6%)^#^	82.58 ± 12.40	1.940^b^	0.123
New Rural Cooperative Medical Insurance	16 (5.4%)^#^	79.19 ± 13.09		
No	2 (0.7%)^#^	76.50 ± 26.16		
Resident Medical Insurance	58 (19.4%)^#^	78.55 ± 11.91		
Hypertension				
No	75 (25.1%)	83.56 ± 11.25	1.596^a^	0.112
Yes	224 (74.9%)	80.91 ± 12.82		
Kidney disease etiology				
Diabetic nephropathy	13 (4.4%)^#^	86.38 ± 12.35	1.092^b^	0.365
Glomerulonephritis	14 (4.7%)^#^	80.00 ± 9.77		
Hypertensive nephropathy	43 (14.4%)^#^	82.74 ± 12.27		
IgA nephropathy	45 (15.1%)^#^	83.16 ± 13.14		
Other	45 (15.1%)^#^	82.51 ± 12.92		
Unknown	139 (46.5%)^#^	80.09 ± 12.44		
Education level				
College or above	137 (45.8%)	82.92 ± 11.39	1.827^b^	0.142
High school	72 (24.1%)	81.81 ± 14.22		
Middle school	68 (22.7%)	78.62 ± 12.67		
Primary school or below	22 (7.4%)	81.59 ± 11.53		
Occupation				
Manual	24 (8.0%)	76.04 ± 15.13	2.098^b^	0.101
Mental laborer	104 (34.8%)	81.28 ± 11.51		
Other	36 (12.0%)	81.31 ± 11.30		
Unemployed	135 (45.2%)	82.86 ± 12.83		
Economic burden				
Low	76 (25.4%)	84.29 ± 12.41	8.695^b^	0.001
Medium	111 (37.1%)	83.52 ± 11.07		
High	112 (37.5%)	77.80 ± 13.01		
Comorbidity score	0.62 ± 0.90		0.056^c^	0.338

^a^ Independent-samples t-test.

^b^ One-way analysis of variance.

^c^ Pearson correlation analysis.

^#^ Percentages may not sum to 100% due to rounding.

### 3.3 Correlation analysis of alexithymia, emotion regulation, psychological resilience, and self-management

Kidney transplant recipients had an mean self-management score of 81.58 ± 12.48. The absolute values of skewness and kurtosis were 0.182 (absolute value < 3) and 0.612 (absolute value < 10), respectively, indicating that the scores did not substantially deviate from a normal distribution (26). As shown in **Table 2**, according to pearson correlation analysis revealed that alexithymia was positively correlated with expressive suppression (r = 0.654, p < 0.001) and negatively correlated with cognitive reappraisal (r = -0.431, p < 0.001), psychological resilience (r = -0.622, p < 0.001), and self-management (r = -0.707, p < 0.001). Cognitive reappraisal showed a positive correlation with self-management (r = 0.493, p < 0.001) and psychological resilience (r = 0.387, p < 0.001). Expressive suppression was negatively correlated with psychological resilience (r = −0.535, p < 0.001) and self-management (r = −0.631, p < 0.001). Self-management and psychological resilience were positively correlated (r = 0.798, p < 0.001).

**Table 2:** Correlation analysis among variables.

Variable		1	2		3	4
			Cognitive reappraisal	Expression Suppression		
1. Alexithymia		1				
2. Emotional regulation	Cognitive reappraisal	-0.431***	1			
	Expression Suppression	0.654***	0.412***			
3. Psychological resilience		-0.622***	0.387***	1	1	
4. Self-management		-0.707***	0.493***	-0.631***	0.798***	1

***p < 0.001.

Given the high correlation between psychological resilience and self-management, we conducted further analysis. Discriminant validity between psychological resilience and self-management was assessed using the heterotrait-monotrait (HTMT) inference test (27). This test evaluates H0: HTMT ≥ 1 against H1: HTMT < 1, where rejection of H0 supporting discriminant validity. A bootstrap procedure with 2,000 resamples yielded a 95% confidence interval of [0.904, 0.987] for the HTMT value. As the upper limit of this interval was strictly less than 1, the null hypothesis was rejected, supporting their retention as separate latent variables in the structural model.

### 3.4 Regression analysis of alexithymia, emotion regulation, psychological resilience, and self-management

As shown in **Supplementary Table 1**, a linear regression analysis was conducted with self-management as the dependent variable and alexithymia, cognitive reappraisal, and psychological resilience as the independent variables. Alexithymia was negatively associated with self-management (β = −0.126, p < 0.001), whereas cognitive reappraisal (β = 0.240, p < 0.001) and psychological resilience (β = 0.559, p < 0.001) were positively associated with self-management. Alexithymia was significantly negatively associated with cognitive reappraisal (β = −0.116, p < 0.001), psychological resilience (β = −0.237, p < 0.001), and self-management (β = −0.126, p < 0.001).

When self-management was treated as the dependent variable (**Supplementary Table 2**), psychological resilience was positively correlated with self-management (β = 0.553, p < 0.001), whereas alexithymia (β = −0.108, p < 0.001) and expressive suppression (β = −0.364, p < 0.001) were negatively correlated with self-management. Alexithymia had a significant negative correlation with self-management (β = −0.108, p < 0.001) and psychological resilience (β = −0.202, p < 0.001), and a significant positive correlation with expressive suppression (β = 0.132, p < 0.001).

### 3.5 Structural equation models of alexithymia, emotion regulation, psychological resilience and self-management

Based on the study hypotheses and literature review, structural equation models were developed to examine the relationships among alexithymia, emotion regulation (cognitive reappraisal and expressive suppression), psychological resilience, and self-management. Model 1 included alexithymia, cognitive reappraisal, psychological resilience, and self-management. The fit indices for Model 1 indicated a satisfactory model fit: χ²/df = 2.784, GFI = 0.911, NFI = 0.933, CFI = 0.955, TLI = 0.944, and RMSEA = 0.077 (**Table 3**). Model 2 included alexithymia, expressive suppression, psychological resilience, and self-management. The fit indices for Model 2 were χ²/df = 3.148, GFI = 0.902, TLI = 0.935, NFI = 0.927, CFI = 0.949, and RMSEA = 0.085. A model is generally considered acceptable when χ²/df < 5 (28).

**Table 3:** Goodness of fit of the structural equation model.

Adaptation index	X^2^/df	GFI	TLI	NFI	CFI	RMSEA
Reference value	≤3	≥0.9	≥0.9	≥0.9	≥0.9	≤0.1
Model 1	2.784	0.911	0.944	0.933	0.955	0.077
Model 2	3.148	0.902	0.935	0.927	0.949	0.085

**Figure 2:**
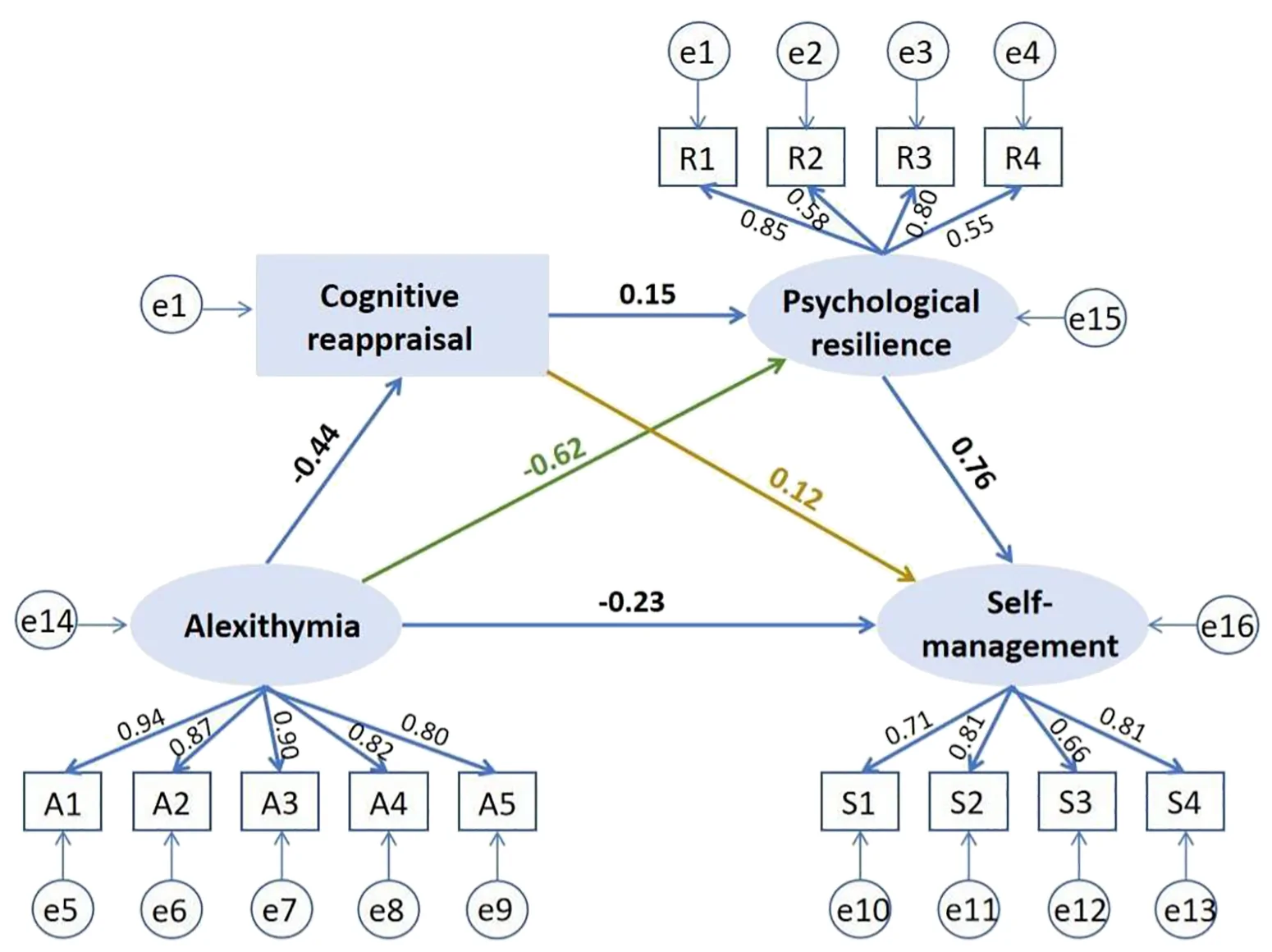
Structural equation model of alexithymia, cognitive reappraisal, psychological resilience and self-management.

The results of Model 1 showed that, with self-management as the dependent variable and alexithymia as the independent variable, the total effect of alexithymia on self-management was −0.453, comprising a direct effect of −0.129 and a total indirect effect of −0.323. The relationship between alexithymia and self-management was partially mediated by cognitive reappraisal (indirect impact = −0.030, 95% CI [−0.053, −0.013] and psychological resilience (indirect effect = −0.265, 95% CI [−0.370, −0.179]. In addition, as shown in **Table 4** and **Figure 2**, the association between alexithymia and self-management was serially mediated by cognitive reappraisal and psychological resilience (indirect effect = −0.028, 95% CI [−0.054, −0.006].

**Table 4:** Direct and indirect effects of alexithymia, cognitive reappraisal and psychological resilience on self-management.

Structural paths	Effect	BootSE	LLCI	ULCI
Direct paths				
Alexithymia →Self-management	-0.129	0.044	-0.221	-0.048
Indirect paths				
Alexithymia →Cognitive reappraisal→Self-management	-0.030	0.011	-0.053	-0.013
Alexithymia →Psychological resilience→Self-management	-0.265	-0.049	-0.370	-0.179
Alexithymia →Cognitive reappraisal→Psychological resilience→Self-management	-0.028	0.011	-0.054	-0.006
Total indirect effect	-0.323	0.049	-0.429	-0.234
Total effect	-0.453	-0.039	-0.539	-0.380

**Figure 3:**
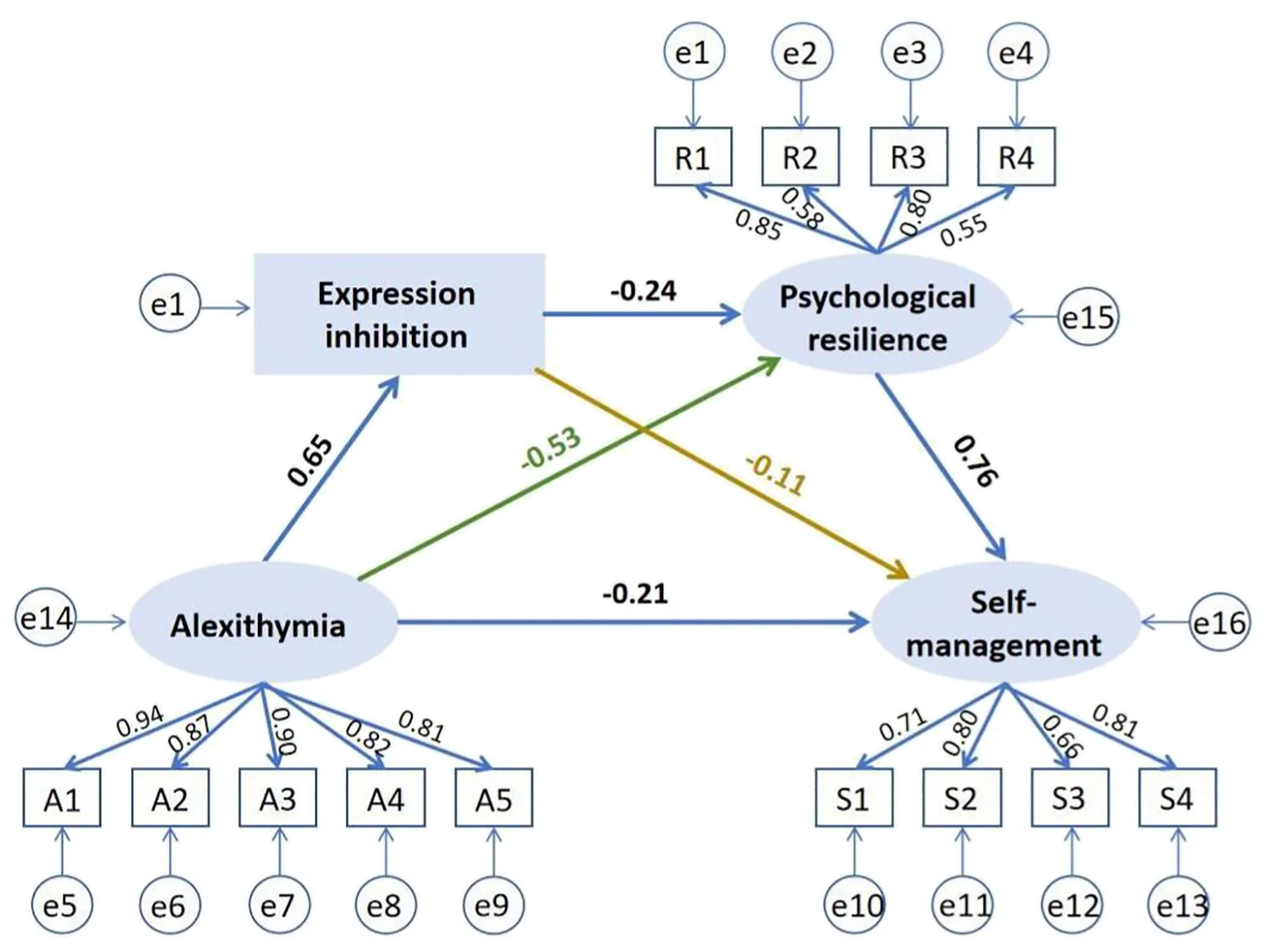
Structural equation model of alexithymia, expressive suppression, psychological resilience and self-management.

The results of Model 2 showed that, with self-management as the dependent variable and alexithymia as the independent variable, the total effect of alexithymia on self-management was −0.456, comprising a total indirect effect of −0.337 and a direct effect of −0.119. The relationship between alexithymia and self-management was partially mediated by expressive suppression (indirect impact = −0.041, 95% CI [−0.079, −0.005]. Additionally, the relationship between alexithymia and self-management was partially mediated by psychological resilience (indirect effect = −0.229, 95% CI [−0.327, −0.150]. Furthermore, as shown in **Table 5** and **Figure 3**, expressive suppression and psychological resilience jointly exerted a serial mediation effect on the relationship between alexithymia and self-management (indirect effect = −0.067, 95% CI [−0.113, −0.028].

**Table 5:** Direct and indirect effects of alexithymia, expressive suppression and psychological resilience on self-management.

Structural paths	Effect	BootSE	LLCI	ULCI
Direct paths				
Alexithymia →Self-management	-0.119	0.043	-0.207	-0.037
Indirect paths				
Alexithymia →Expression Suppression→Self-management	-0.041	0.018	-0.079	-0.005
Alexithymia →Psychological resilience→Self-management	-0.229	0.047	-0.327	-0.150
Alexithymia →Expression Suppression→Psychological resilience→Self-management	-0.067	0.021	0.113	-0.028
Total indirect effect	-0.337	0.047	-0.441	-0.256
Total effect	-0.456	0.039	-0.544	-0.384

## 4 Discussion

Based on stress and coping theory, this study found that alexithymia was both directly and indirectly associated with self-management, with emotion regulation and psychological resilience serving as significant mediators. To the best of our knowledge, this is the first study to elucidate the underlying mechanisms connecting alexithymia and self-management in kidney transplant recipients, thereby providing empirical evidence and potential intervention targets for developing tailored psychological interventions to enhance self-management in this population.

We found that alexithymia was significantly associated with poorer self-management in kidney transplant recipients, consistent with Calia et al. (9). Among the core dimensions of emotional processing deficits associated with alexithymia, the negative dimension, namely difficulty identifying and describing distressing feelings, appeared to exert the most pronounced effect. After transplantation, recipients often experience coexisting psychological and somatic discomforts, such as depression, anxiety and fatigue, making it difficult for them to clearly distinguish and articulate these experiences. As a result, problems may arise in dietary management, including emotional eating and insufficient intake of high-quality protein. In treatment management, an inability to express negative emotions related to medication side effects may reduce medication adherence and may even lead some patients to attempt to alleviate their symptom burden by reducing immunosuppressant use (29–32). By contrast, difficulty identifying and describing positive feelings mainly affected psychosocial management and physical activity management. If patients are unable to perceive and express positive changes during recovery, such as changes in self-perception, post-traumatic growth or a greater appreciation of life, they may be less able to use positive emotions to strengthen their confidence in rehabilitation, thereby reducing the continuity and initiative of physical activity (33, 34). At the same time, barriers to the expression of positive emotions may make it difficult for recipients to obtain social support, further weakening psychosocial adaptation and initiative in social activities, and potentially placing their social support system in a marginalized position (35). The externally oriented thinking dimension of alexithymia may also impair self-management by leading to passive treatment compliance and inflexible health behaviors. These findings suggest that nursing staff could consider developing individualized interventions tailored to the specific features of alexithymia. For patients with difficulty identifying and describing feelings, meditation, emotion and body awareness exercises may help strengthen the differentiation and linkage between bodily signals and emotions. For those with marked externally oriented thinking, cognitive reappraisal and situational exposure exercises may be used to encourage them to record their experiences related to medication use, thereby shifting attention from worries about side effects to internal bodily sensations and the sense of stability after taking medication (36). Family empowerment and the development of social support should also be strengthened. External support may be provided through family education and peer support programs, while family members should be guided to recognize changes in the patient’s emotional state and to encourage medication adherence in a supportive rather than blaming manner. Whether alleviating alexithymia through these approaches can improve self-management and transplant outcomes warrants investigation in future studies (37).

The association between alexithymia and self-management was mediated by expressive suppression and cognitive reappraisal. Owing to impaired emotional processing, patients with alexithymia may struggle to engage in adaptive positive emotion regulation strategies and may instead rely on expressive suppression to avoid emotional experiences, thereby weakening the initiative required for self-management behaviors such as treatment adherence and dietary control (38). By contrast, cognitive reappraisal may buffer the negative association of alexithymia by reconstructing the patient’s interpretation of disease-related events, helping to foster a more positive rehabilitation mindset and improve self-management efficacy (39). The association between alexithymia and emotion regulation deficits has also been observed in other populations. Goetz et al. found that alexithymia was associated with eating disorder symptoms through difficulties in immediate emotion regulation (40). Similar patterns, including elevated alexithymia levels, excessive use of suppression strategies, and impaired cognitive reappraisal, have also been reported in individuals with panic disorder (41). The interplay between alexithymia and maladaptive emotion regulation may represent a transdiagnostic mechanism that impairs disorder-specific self-management behaviors across different patient populations. These findings indicate that nursing staff may consider early identification of alexithymia and assessment of patients’ emotion regulation tendencies. Through cognitive restructuring training, patients may be guided to adopt cognitive reappraisal and reduce their reliance on expressive suppression. For example, patients may be encouraged to reframe medication taking as “an active means of taking control of life” and to record experiences of stability instead of negative anticipatory imagery. In addition, using emotion cards to record daily mood states and encouraging patients during follow-up to describe the sources of their emotions may gradually reduce internal avoidance. These strategies may help improve adherence and initiative in self-management and contribute to a better long-term prognosis.

Psychological resilience also significantly mediated the association between alexithymia and self-management, consistent with Hu et al. (42). Among adolescents with depression, alexithymia also indirectly influences non-suicidal self-injurious behavior through the mediating effect of psychological resilience, thereby providing empirical support for the cross-population applicability of psychological resilience as a mediating variable (43). Alexithymia may be indirectly associated with poorer self-management through lower psychological resilience, whereas higher resilience may attenuate this negative association, possibly by mobilizing internal coping resources (13). This may help recipients cope more actively with post-transplant rehabilitation challenges and to engage more proactively in self-management behaviors, including dietary control, treatment adherence, physical activity, and psychosocial adjustment. Nursing staff may adopt acceptance and commitment therapy to guide patients in observing physical discomfort with openness and to reduce avoidance or oppositional responses to symptoms (44). At the same time, patients may be helped to clarify their personal values, such as “maintaining physical function to return to normal life”, and to transform daily medication taking into an action commitment aligned with these values. Such interventions may help patients maintain health behaviors despite discomfort, potentially weakening the negative link between alexithymia and self-management.

Alexithymia was associated with self-management in kidney transplant recipients through the serial mediating effects of emotion regulation strategies (cognitive reappraisal and expressive suppression) and psychological resilience. Existing evidence suggests that deficits in emotional identification may increase reliance on expressive suppression while weakening cognitive reappraisal, thereby depleting psychological resources (45). This imbalance in emotion regulation may be related to reduced activation of psychological resilience and may reduce the psychological resources available for coping with disease recover (39). Insufficient psychological resilience may ultimately weaken the initiative and persistence required for self-management, thereby compromising the implementation of rehabilitation behaviors such as dietary control and treatment adherence (46). Helping patients who tend to rely on expressive suppression to shift toward cognitive reappraisal may allieve the negative impact of alexithymia on self-management by enhancing psychological resilience. These findings suggest that routine screening for alexithymia and emotion regulation strategies could be considered in follow-up outpatient care for kidney transplant recipients, and brief, structured cognitive reappraisal training programs may be explored. A study by Kam et al. (47) demonstrated that even a brief 2-week cognitive reappraisal training program can increase individuals’ confidence in successfully using cognitive reappraisal and modify their emotion regulation strategies. Nursing staff may instruct patients to record one negative event related to disease management each day, such as mood fluctuations after medication or physical discomfort affecting work, and then attempt to reframe its meaning. For example, reframing “I cannot work” as “my body is adapting, and I need to adjust my pace”. expressive suppression to cognitive reappraisal. This approach may facilitate a shift from expressive suppression to cognitive reappraisal. This approach may help activate psychological resilience and strengthen coping resources, which could contribute to improved self-management.

The high correlation between psychological resilience and self-management (r = 0.798) warrants discussion. Although the HTMT test supported their discriminant validity (95% CI [0.904, 0.987]), this strong association may reflect conceptual overlap, particularly between the “medical management” dimension of the resilience scale and the “treatment management” dimension of the self-management scale. However, resilience represents an internal coping resource, whereas self-management encompasses external health behaviors. The strong association may also reflect a bidirectional relationship: resilience facilitates self-management, and successful self-management may in turn reinforce resilience. Future studies should further examine the distinctiveness of these constructs through item-level confirmatory factor analysis. The total effect of alexithymia on self-management differed slightly between Model 1 (−0.453) and Model 2 (−0.456), as did the direct effect (−0.129 vs. −0.119). This minor discrepancy is expected when the same independent variable–dependent variable relationship is decomposed through different mediating pathways in separate models, as the indirect effects captured by each mediator differ in magnitude. The consistency of the overall pattern across both models—namely, that alexithymia was both directly and indirectly associated with self-management—supports the robustness of our findings.

### 4.1 Limitations

This study has several limitations. First, the cross-sectional design precluded determination of the temporal ordering and causal relationships among the variables, and the path relationships identified by model fitting require further verification and refinement in future longitudinal studies. Second, all measures were self-reported, which may introduce recall bias and common method variance. Although Harman’s single-factor test suggested that common method bias did not greatly affect the findings, the possibility of inflated associations cannot be entirely excluded. Third, the high correlation between psychological resilience and self-management (r = 0.798) raises the possibility of construct overlap. Although the HTMT inference test supported their discriminant validity, the strong association may have influenced the magnitude of the indirect effect estimates for the resilience mediation pathway, which was the largest indirect effect in both models. Future studies should further examine the distinctiveness of these constructs, potentially through confirmatory factor analysis with item-level data. Fourth, the generalizability of our findings may be limited. Data were collected from three tertiary hospitals in China, and cultural norms regarding emotion regulation may differ across populations. Future studies should recruit more diverse samples across different cultural contexts to validate our findings. Fifth, cognitive reappraisal and expressive suppression were examined in two separate models rather than a single integrated model. This approach prevented direct comparison of their relative contributions while controlling for the other strategy. Future studies could consider incorporating both strategies within a single model to assess their unique and joint mediating effects. Sixth, several clinically relevant confounders were not controlled for in the model. Time since transplantation was only examined descriptively. Immunosuppressant regimens were not included as covariates. These factors may affect both psychological functioning and self-management. Future studies should include these variables as covariates to reduce potential confounding.

## 5 Conclusion

Based on stress and coping theory, this study constructed a structural equation model incorporating alexithymia, emotion regulation, psychological resilience, and self-management. The findings showed that alexithymia was both directly and indirectly associated with self-management in kidney transplant recipients. Additionally, alexithymia indirectly affected self-management through emotion regulation and psychological resilience. Healthcare professionals may help weaken the negative association between alexithymia and self-management by assisting kidney transplant recipients in modifying their emotion regulation strategies and enhancing psychological resilience, thereby increasing psychological resource reserves, strengthening coping capacities, and improving self-management.

## Data Availability

The raw data supporting the conclusions of this article will be made available by the authors, without undue reservation.
