# Supplementary material for: The relationship between alexithymia, emotion regulation, psychological resilience, and self-management behaviors in kidney transplant recipients: a multicenter structural equation modeling study
Source: Front Psychiatry. 2026 Aug 17;17:1909478. doi: 10.3389/fpsyt.2026.1909478 (PMC13528302; doi:10.3389/fpsyt.2026.1909478)
Supplement: Supplementary file 1 [file SupplementaryFile1.docx]

Supplementary Material

**Table S1** Regression analysis of alexithymia, cognitive reappraisal, psychological resilience and self-management

| **Result variables** | **Prediction variable** | **β** | **t** | **R²** | **F** |
| --- | --- | --- | --- | --- | --- |
| Cognitive reappraisal |  |  |  | 0.186 | 67.702 |
|  | Alexithymia | -0.116 | -8.228*** |  |  |
|  |  |  |  |  |  |
| Psychological resilience |  |  |  | 0.404 | 100.342 |
|  | Alexithymia | -0.237 | -11.245*** |  |  |
|  | Cognitive reappraisal | 0.230 | 2.930** |  |  |
|  |  |  |  |  |  |
| Self-management |  |  |  | 0.727 | 262.309 |
|  | Alexithymia | -0.126 | -7.343*** |  |  |
|  | Cognitive reappraisal | 0.240 | 4.424*** |  |  |
|  | Psychological resilience | 0.559 | 14.120*** |  |  |

***p* < 0.01,****p* < 0.001

**Table S2** Regression analysis of alexithymia, expressive suppression, psychological resilience, and self-management

| **Result variables** | **Prediction variable** | **β** | **t** | **R²** | **F** |
| --- | --- | --- | --- | --- | --- |
| Expression Suppression |  |  |  | 0.427 | 221.658 |
|  | Alexithymia | 0.132 | 14.888*** |  |  |
|  |  |  |  |  |  |
| Psychological resilience |  |  |  | 0.416 | 105.270 |
|  | Alexithymia | -0.202 | -8.091*** |  |  |
|  | Expression Suppression | -0.474 | -3.825*** |  |  |
|  |  |  |  |  |  |
| Self-management |  |  |  | 0.725 | 259.690 |
|  | Alexithymia | -0.108 | -5.669*** |  |  |
|  | Expression Suppression | -0.364 | -4.157*** |  |  |
|  | Psychological resilience | 0.553 | 13.762*** |  |  |

****p* < 0.001
